# Music-induced cortical plasticity: Protocol for a systematic review

**DOI:** 10.1371/journal.pone.0336011

**Published:** 2026-07-02

**Authors:** Isaiah Osei Duah Junior, Danielle S. Rodriguez, Cassandra M. Germain

**Affiliations:** 1 Department of Psychology, John R. and Kathy R. Hairston, College of Health and Human Sciences, North Carolina Agricultural and Technical State University, Greensboro, North Carolina, United States of America; 2 Department of Optometry and Visual Science, College of Science, Kwame Nkrumah University of Science and Technology, Kumasi, Ghana; Ceuma University: Universidade Ceuma, BRAZIL

## Abstract

Music engages sensory, motor, cognitive, and emotional systems, making it a powerful model for studying experience-dependent neuroplasticity. Although research on music-related brain changes is expanding, integration of structural, functional, and cerebrovascular findings remains limited, and effects on higher-order cognitive processes remain unclear. This systematic review will primarily synthesize evidence on music-induced cortical adaptations, including structural changes (e.g., gray and white matter alterations), functional modifications in neural networks, and cerebrovascular dynamics. Further, associations with behavioral measures such as attentional control, executive functioning, and language processing will also be examined when these outcomes are directly linked to the neural adaptations across the adult lifespan (≥ 50 years). The protocol was prepared in accordance with the Preferred Reporting Items for Systematic Reviews and Meta-Analysis Protocols (PRISMA-P) guidelines and has been indexed in International Prospective Register of Systematic Reviews (PROSPERO ID: CRD420251159362). The systematic review will be conducted and reported in accordance with the Preferred Reporting Items for Systematic Reviews and Meta-Analysis (PRISMA) guidelines. Using controlled vocabularies and medical subject headings (MeSH), a systematic search will be conducted across PubMed, Scopus, Web of Science, PsycINFO, EMBASE, CENTRAL (Cochrane Central Register of Controlled Trials), and Google Scholar databases, and supplemented by citation tracking from relevant review articles. Eligible studies will include randomized controlled trials (RCTs), non-randomized controlled trials, quasi-experimental, and observational designs (longitudinal, case-control, cross-sectional studies). The primary outcome will be changes in brain structure, function, and cerebrovascular hemodynamic activity following music intervention. Cognitive measures will be reviewed and consolidated as secondary endpoints. Data extraction, risk of bias and quality assessment will be performed independently by two reviewers using validated instruments, including Cochrane Risk of Bias 2 (RoB 2.0), Risk Of Bias In Non-randomized Studies – of Interventions (ROBINS-I), Risk Of Bias In Non-randomized Studies of Exposures (ROBINS-E), Strengthening the Reporting of Observational Studies in Epidemiology (STROBE), and National Heart, Lung, and Blood Institute (NHLBI) tools. A narrative synthesis will be conducted in accordance with Synthesis Without Meta-analysis (SWiM) guidelines, with meta-analyses undertaken where appropriate. Certainty of evidence will be assessed using Grading of Recommendations Assessment, Development, and Evaluation (GRADE) scale. Collectively, this protocol establishes a rigorous framework to systematically evaluate how music shapes the brain’s structure, function, and vascular systems, and how these changes translate into cognitive and behavioral outcomes.

## Introduction

Music is a unique human activity that engages the brain in a uniquely multimodal manner, simultaneously recruiting sensory, motor, cognitive, and affective systems [1–5]. Beyond its cultural and aesthetic significance, music has emerged as a powerful model for studying neuroplasticity, the brain’s capacity to reorganize its structure, connectivity, and function in response to experience [6–9]. By activating auditory, visual, motor, cognitive, and emotional networks in parallel [1–4,10–13], music provides a rich, multisensory stimulus for probing experience-dependent neural plasticity across the lifespan [14–18].

Although evidence for the cognitive benefits of music exposure (e.g., intervention, therapy, training) remains mixed [19–21], converging neurobiology data consistently demonstrate structural and functional cortical adaptations associated with musical training and engagement [22–29]. Structurally, music exposure has been linked to increased gray matter volume and cortical thickness in sensory and motor cortices, enhanced white matter integrity in fiber tracts such as the corpus callosum and arcuate fasciculus, and region-specific cortical map reorganization [22–27]. Functionally, music engagement supports enhanced auditory discrimination [28–32], improved sensorimotor integration [33–35], refined executive function [36], and strengthened intra- and inter-network connectivity [37–39]. These functional improvements, together with anatomical adaptations, have been observed across the lifespan, suggesting music may promote cognitive resilience [22–28,31,36,40,41,42]. Conversely, a large-scale multi-site study have reported no association between musical training and early auditory neural responses, such as brainstem-level frequency-following response measures, suggesting that music-related plasticity may preferentially manifest at higher-order, context-dependent stages of auditory and cognitive processing rather than at subcortical encoding levels [43].

In addition to structural and functional adaptations, emerging evidence suggests that music engagement may influence cerebral blood flow (CBF), a critical physiological marker of cortical activity and plasticity [44–46]. The CBF reflects the brain’s metabolic demands and vascular responsiveness [47], providing a dynamic index of localized neural activity that supports synaptic remodeling [48]. Despite its importance, the relationship between music-induced plasticity and CBF remains poorly understood, leaving the vascular and hemodynamic dimensions understudied. Systematic reviews that integrate CBF metrics with measures of cortical plasticity are particularly promising, as they can consolidate fragmented findings, reveal consistent patterns across diverse study designs, and provide mechanistic insights linking neural remodeling to functional and metabolic dynamics. By simultaneously engaging sensorimotor networks, music has the potential to modulate regional cortical perfusion, enhance neurovascular coupling, and facilitate experience-dependent cortical reorganization [41,49,50].

**Fig 1:**
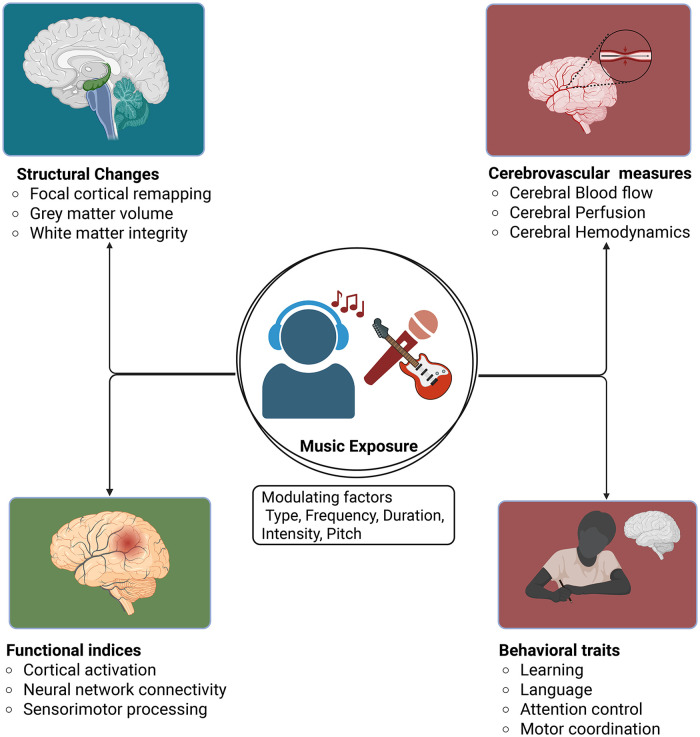
Multidimensional effects of music exposure on the brain and behavior. The figure illustrates how music exposure influences the brain across multiple domains: in the top left, music drives focal cortical remapping, alters grey matter volume, and enhances white matter integrity, reflecting its capacity to reshape neural architecture; in the bottom left, functional indices are depicted, including changes in cortical activation, neural network connectivity, and sensorimotor processing; in the top right, the diagram highlights cerebrovascular measures, emphasizing music’s effects on cerebral blood flow, perfusion, and hemodynamics, which together support healthier vascular function and metabolic activity in the brain; and in the bottom right, shifts in behavioral and cognitive domains are shown, encompassing learning, language, attention control, and motor coordination. Created in BioRender. Osei Duah, I. (2025) https://BioRender.com/w0phamk.

Of note, cognitive and behavioral findings also complement these neural evidence, with music training linked to improved performance in language acquisition [31,41,42], verbal memory [51–53], attentional control [54,55], short term memory [56], emotional regulation [57,58], social communication [59,60], and motor coordination [61]. Additionally, music exposure has been associated with improved academic performance [62], enhance cognitive function and reduce cognitive decline [63,64]. Collectively, these findings illustrate how music-induced cortical plasticity translate into behavioral outcomes, yielding meaningful practical benefits (see Fig 1).

Despite notable scientific advances in this field, the evidence base remains highly fragmented, primarily due to marked heterogeneity in study populations, intervention protocols, and assessment methodologies. For example, some studies focus exclusively on adults or children [22,25,29,65,66], others include both musicians and non-musicians [66–71], and still others examine the short- and long-term effects of music-based interventions [29,66,72,73]. The modalities of music exposure and engagement under study are diverse, ranging from actively playing instruments and singing to passive listening, and structured music therapies [74–76]. In addition, neural outcomes have been characterized using a broad spectrum of techniques, including diffusion tensor imaging (DTI) [77,78], electroencephalography (EEG) [79–81], magnetoencephalography (MEG) [82–84], and structural and/or functional magnetic resonance imaging (MRI) [25,74,85–88]. This methodological and conceptual heterogeneity complicates interpretation and constrains cross-study comparability. Taken together this impedes the formulation of generalizable conclusions about the nature, extent, and persistence of music-induced cortical plasticity, as well as its translational potential for therapeutic innovation.

Existing reviews and meta-analyses on neuromusicology have provided valuable insights but remain limited in scope. Most have focused primarily on music’s effects within auditory and motor cortices, regions central to perception and production [1,89]. Yet emerging evidence indicates that music also induces plasticity in occipital [90], prefrontal [91], parietotemporal [92,93], and limbic cortices [94]. These higher-order regions support multisensory integration, executive function, attention, and emotional regulation [95,96]. Collectively, these findings provide convergent evidence that musical training is associated with structural remodeling, functional network refinement and efficiency, and cerebrovascular adaptations. However, no systematic review has yet comprehensively synthesized music’s influence across these cortical domains. Prior reviews disproportionate emphasize behavioral and cognitive outcomes, such as memory [97,98], attention [99,100], language [101,102], and emotional regulation [57,103]. Although informative, these behavioral outcomes offer only indirect insight into the neural mechanisms through which music exerts its effects. In the absence of systematic integration across structural, functional, and cerebrovascular findings, the neurobiological underpinnings of music-induced behavioral changes will remain poorly understood. Addressing this evidence gap is critical, as delineating cortical adaptations offers direct mechanistic insight and advances the field beyond purely descriptive accounts of music influence on behavior.

Clarifying the neurobiological underpinning of music carries both theoretical and practical significance. Systematic mapping of how music reshapes cortical architecture, connectivity, and cerebrovascular remodeling advances fundamental principles of neuroplasticity while informing translational innovation. If music reliably induces plasticity in neural circuits supporting sensory integration, executive function, attention, or emotion regulation, it can be leveraged to develop novel, neuroscience-based interventions. Its cultural ubiquity, appeal, and reach render music uniquely suited as a potential treatment strategy for neurological and psychiatric conditions, including stroke, dementia, depression, neurodevelopmental, and neurodivergent disorders.

Collectively, these considerations underscore the need for a systematic review that critically consolidates evidence on music-related cortical plasticity. By applying transparent, rigorous, and reproducible methods, the present review will synthesize findings across structural and functional domains, examines effects spanning auditory, motor, visual, and higher-order associative cortices, and situates these outcomes within emerging mechanistic frameworks. This integrative approach aims to clarify inconsistencies in the literature, identify methodological strengths and limitations, and highlight critical gaps for future investigation. The primary objective of this systematic review is to comprehensively synthesize evidence on music-related neuroplasticity. Specifically, the review will focus on three domains of neural adaptation: (i) structural brain measures, including cortical thickness and gray and white matter integrity; (ii) functional brain organization and neural activity, such as task-based and resting-state functional connectivity; and (iii) cerebrovascular adaptations, including regional cerebral blood flow and neurovascular coupling. Further, the secondary aim is to synthesize evidence linking these neural measures to behavioral outcomes associated with music engagement or musical training, thereby elucidating the functional relevance of observed neuroplastic changes. Interpretation of the findings is guided by an explicit theoretical framework in which musical training is conceptualized as a multimodal, enriched experience that repeatedly co-engages auditory, motor, cognitive, emotional, and reward networks. Altogether, such sustained co-activation is hypothesized to promote experience-dependent plasticity through established processes, including synaptic strengthening, large-scale network reorganization, and neurovascular adaptation (see **Fig 1**).

## Materials and methods

This systematic review protocol was prepared following the PRISMA-P checklist (S1 File) to provide a structured framework for the design, planning, and prospective reporting of methods [104,105]. The protocol is prospectively indexed in PROSPERO (Protocol registration ID: CRD420251159362) to ensure methodological rigor, prevent unnecessary duplication, and promote accountability and transparency in the review process. The systematic review will be conducted in accordance with the PRISMA guidelines to ensure comprehensive, transparent, and standardized reporting [106]. In instances where quantitative synthesis or meta-analysis is not feasible due to heterogeneity in study designs, populations, interventions, or outcomes, the SWiM reporting framework will be applied to guide the narrative synthesis and enhance the clarity, transparency, and reproducibility of the evidence synthesis process [107].

### Eligibility criteria

#### Inclusion criteria.

The eligibility criteria for this systematic review will be defined according to the PICOS framework, encompassing the population, intervention, comparators, outcomes, and study design.

**Population (P):** This review will include studies enrolling participants aged ≥ 50 years without formal musical training. Studies enrolling infants, children, and adolescents will be excluded due to potential brain maturation influences that may confound interpretation of music-induced adaptations. Both healthy and clinical populations (e.g., individuals with neurodevelopmental or neurodegenerative conditions) will be eligible, provided structured music exposure (e.g., intervention, therapy or training) is part of the intervention. Studies with formal musicians will also be excluded due to potential confounding effects.

**Intervention/Exposure (I):** The review will include studies that evaluate different forms of music exposure (see **Table 1** for a comprehensive list), including but not limited to active music making (instrumental practice, vocal training, structured lessons, ensemble participation etc.), and passive music listening, such as background and attentive listening. Both short-term and long-term music exposures will be considered, as well as observational studies examining the extent and type of musical experience.

**Table 1:** Search strategy developed to be adapted for all databases.

PICOS Element	Concepts	Keywords
1	Music measures	music* OR musical OR music training OR music making OR music education OR music therapy OR music practice OR musical experience OR music intervention OR music listening
2	Neuroplasticity measures	Structural brain adaptations OR morphological brain alterations OR neuroanatomical changes OR brain morphology indices OR gray matter density OR gray matter volume OR white matter organization OR white matter integrity OR cortical thickness OR cortical architecture OR neurostructural markers OR brain connectivity patterns OR neural circuitry reorganization OR cerebral blood flow OR brain blood flow OR cerebral perfusion OR brain perfusion OR regional cerebral blood flow OR cerebral circulation OR brain circulation OR cerebral hemodynamics OR cerebral vascular flow OR cerebral microcirculation OR cerebral oxygenation OR cerebral autoregulation OR neural processing improvements OR cognitive function enhancements OR perceptual gains OR information-processing efficiency OR sensorimotor integration
3	Combine	Search: (#1) and (#2)

Boolean operators are used to structure search queries: use ‘OR’ to link keywords within the same concept, capturing synonyms or related terms, and use ‘AND’ to combine keywords across different concepts, ensuring that results include all specified concepts.

**Comparison (C):** Studies will be eligible if they employ cross-sectional, longitudinal, randomized, or quasi-experimental designs in which there is a control arm consisting of participants receiving no music exposure.

**Outcomes (O):** The primary outcome of interest will encompass structural brain adaptations, functional and neurovascular changes. Structural outcomes include morphological brain alterations, neuroanatomical changes, brain morphology indices, gray matter density, gray matter volume, white matter organization and integrity, cortical thickness and architecture, neurostructural markers, brain connectivity patterns, and neural circuitry reorganization. Functional outcomes include neural processing improvements, cognitive function enhancements, perceptual gains, information-processing efficiency, and sensorimotor integration. Neurovascular outcomes will comprise measures of cerebral blood flow, oxygenation, and autoregulatory function. The secondary outcomes of this study will include cognitive domains such as working memory, language, attention control, and executive functions, as well as broader behavioral and cognitive performance measures, including learning efficiency, skill acquisition, academic achievement, social competence, cognitive-behavioral outcomes, and adaptive functioning. Of note, the latter will be extracted and consolidated only when the study has reported one of the primary measures.

**Study design (S):** The review will include a broad range of study designs to capture the diverse evidence on music-related plasticity and brain outcomes. These will include cross-sectional studies, case-control studies, quasi-experimental, and non-randomized controlled trials comparing individuals with and without music exposure or intervention, to examine group differences in neural plasticity as well as cognitive and behavioral outcomes to explore associations between musical experience and markers of brain structure, brain function, and cerebrovascular hemodynamic activity. The review will also include longitudinal intervention studies, where participants undergo structured music training or music-based activities over a defined period with both pre- and post-intervention assessments, providing insights into causal effects of music engagement on neural plasticity and cognitive or behavioral change. In addition, RCTs which is regarded as the gold standard for causal inference, will be incorporated where music-based interventions such as instrumental training, singing, ensemble participation, or music interventions are compared to active or passive control groups such as non-musical enrichment, leisure activities, or no intervention, thereby helping to determine the specificity and robustness of music-related effects. The review will further include neuroimaging studies employing modalities such as functional magnetic resonance imaging (fMRI), electroencephalography (EEG), electroretinography (ERG), Event-Related Potentials (ERP), magnetoencephalography (MEG), diffusion tensor imaging (DTI), or structural MRI when they examine the structural and functional correlates of music engagement. These studies may adopt either cross-sectional or longitudinal designs but must explicitly assess neural outcomes linked to music training or experience. By including this variety of designs, the review will capture both correlational and causal evidence, spanning behavioral and neurobiological levels of analysis.

#### Exclusion criteria.

The review will exclude studies if they involve human participants under eighteen years (given the temporal changes in brain development), non-human primates, as this review focuses exclusively on human participants from young adulthood to older adults. Research that does not include music engagement or where the effects of music cannot be independently assessed, such as interventions combined with other modalities without isolating music-specific contributions, will also be excluded. Additionally, studies lacking a comparison group or appropriate control conditions will not be considered, as these designs do not allow for the evaluation of causal or relative effects of music engagement. Studies will be excluded if they do not report measurable outcomes related to structural or functional neuroplasticity, cognitive functions, or behavioral and psychosocial domains associated with music engagement; those reporting only subjective experiences or anecdotal observations will similarly be excluded. Finally, publication types such as reviews, commentaries, editorials, conference abstracts without full datasets, methodological or theoretical papers without original empirical findings, and non-peer-reviewed or unpublished studies without sufficient data will be excluded. These criteria ensure that only high-quality, empirically rigorous studies providing meaningful evidence on the effects of music engagement are included.

### Database sources and search strategy

A comprehensive search will be conducted in electronic databases including PubMed, Scopus, Web of Science, PsycINFO, EMBASE, Google Scholar, and the CENTRAL from inception to the present, with no restrictions on language or publication year. Additional sources will include reference lists of eligible studies and pertinent reviews. Search terms will be derived from the PICOS framework and will consist of combinations of keywords and controlled vocabulary (e.g., MeSH terms) related to music exposure, training, or engagement; neuroplasticity, brain structure, brain function, or cognitive outcomes; and human participants. Boolean operators (“AND,” “OR”) will be used to combine terms (see **Table 1**).

### Study selection

**Fig 2:**
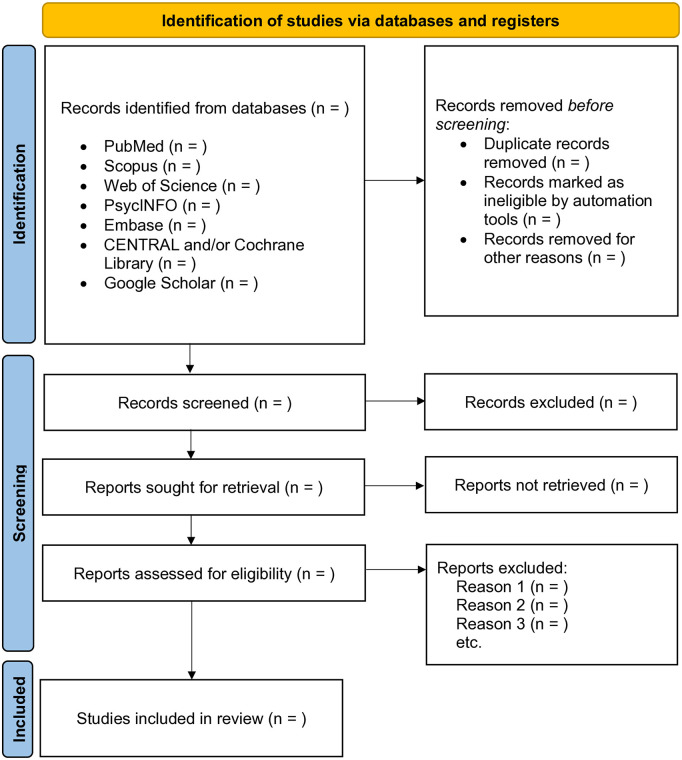
PRISMA flow diagram for study selection. The PRISMA flow diagram outlines the study-selection protocol for a systematic review, documenting each step from identification of records across major databases through screening and eligibility assessment to final study inclusion.

The studies that will be retrieved from the databases will be organized by database using the “My Groups” feature in EndNote Reference Manager Version 20 and subsequently exported to Covidence for comprehensive screening based on a priori eligibility criteria. Specifically, two reviewers (I.O.D.J. and D.S.R.) will independently screen all titles and abstracts to determine their relevance to the research question. Studies that appear to meet the inclusion criteria, or those for which the abstract provides insufficient information to decide, will be retrieved in full text for further evaluation. Each full-text article will then be assessed independently by the two reviewers against the pre-specified inclusion and exclusion criteria. Any discrepancies or disagreements between the reviewers at either the screening or full-text review stage will be resolved through discussion and consensus. If consensus cannot be reached, a third reviewer (C.M.G.) will be consulted to make a final decision. The entire study selection process, including the number of records identified, screened, excluded, and included, will be systematically documented using a PRISMA flow diagram to ensure transparency and reproducibility (see **Fig 2).**

### Data extraction

Data will be independently extracted by two reviewers (I.O.D.J. and C.M.G.) using a standardized extraction form. The extracted information will include study characteristics such as author, year, country, study design, sample size, age range, and population. Details of the intervention or exposure will be collected, including the type, duration, frequency, and intensity of music engagement or training. Comparison conditions will be recorded, including the type of control group or alternative activity. Outcome measures will encompass neuroplasticity indicators (structural, functional, and cerebrovascular) together with cognitive and behavioral performance metrics, and assessment methods. The key findings and statistical results will be extracted and synthesized. Any discrepancies in data extraction will be resolved through consensus or, if necessary, with input from a third reviewer.

### Risk of bias

The risk of bias of included studies will be assessed independently by two reviewers (I.O.D.J. and D.S.R.), with any disagreements resolved through discussion or consultation with a third reviewer (C.M.G.). The assessment tools will be selected according to study design to ensure a rigorous evaluation. For RCTs, the RoB 2.0 will be used to evaluate potential biases across key domains, including the randomization process, deviations from intended interventions, missing outcome data, outcome measurement, and selection of reported results, with each domain rated as “low risk,” “some concerns,” or “high risk,” and an overall risk-of-bias judgment assigned for each study [108]. For non-randomized interventional studies, the ROBINS-I tool will be used to assess bias across domains such as confounding, selection of participants, classification of interventions, deviations from intended interventions, missing data, outcome measurement, and selection of reported results [109]. Similarly, the ROBINS-E will be used to assess bias in observational studies by examining exposure effects across multiple domains, including confounding, participant selection, exposure classification, deviations from intended exposures, missing data, outcome measurement, and selective reporting, rating each as “low” “some concerns,” or “high” risk, with an overall judgment provided for the study [110]. For observational studies, adherence to the STROBE checklist will be adopted to guide the evaluation of study quality and reporting transparency [111].

### Quality assessment

The methodological quality of the studies included in the review will be assessed using the NHLBI quality assessment tools to evaluate overall study rigor (See link: https://www.nhlbi.nih.gov/health-topics/study-quality-assessment-tools). These assessments will consider the clarity of research questions, definition and selection of study populations, sample size justification, validity and reliability of exposure and outcome measurements, appropriateness of statistical analyses, adjustment for confounding variables, and transparency in reporting, with studies classified as “good”, “fair”, or “poor” based on the overall evaluation. For neuroimaging studies, an adapted set of quality assessment criteria will be applied to account for the unique methodological considerations of this research. These criteria will focus on sample size adequacy, participant selection and description, imaging modality and acquisition parameters, preprocessing and analysis pipelines, statistical rigor, correction for multiple comparisons, and clarity of reporting, with studies rated according to methodological rigor and susceptibility to bias. Taken together, this structured, design-specific approach will ensure a comprehensive evaluation of study quality, enhance the reliability of the evidence synthesis and support a robust interpretation of findings.

### Data synthesis

A narrative synthesis will be conducted in accordance with the SWiM guidelines due to the anticipated heterogeneity across included studies in terms of study design, intervention characteristics, and outcome measures [107]. The narrative synthesis will involve a structured and systematic approach to summarizing findings across studies, highlighting patterns, consistencies, and discrepancies in results. Where appropriate, results will be presented in tables, with separate consideration given to the type of study design, as well as to those employing active or passive music exposure. Further, where feasible, effect sizes, such as Cohen’s d or Hedges’ g, will be extracted from primary studies or calculated from reported statistics including means, standard deviations, t-values, or F-values to allow for quantitative comparison across studies. Structural and functional neuroplasticity outcomes will be summarized according to brain region (e.g., prefrontal cortex, motor cortex, auditory regions, hippocampus), modality (e.g., structural MRI, DTI, fMRI, EEG, ERG, ERP, Eye tracking e.t.c), and measurement method (e.g., voxel-based morphometry, cortical thickness analysis, connectivity metrics, hemodynamic or electrophysiological indices). Behavioral and cognitive outcomes will be categorized by domain to facilitate interpretation, including memory (working memory, episodic memory), executive function (inhibitory control, cognitive flexibility, planning), attention and processing speed, social and emotional skills (emotional regulation, empathy, interpersonal functioning), and learning and academic performance. Where subsets of studies demonstrate sufficient homogeneity in intervention type, outcome measures, and study design, meta-analyses will be conducted to quantitatively synthesize results [112]. Heterogeneity among studies will be assessed using the I² statistic, with thresholds interpreted as low (25%), moderate (50%), and high (75%) heterogeneity, and random-effects models will be applied to account for expected between-study variability. Sensitivity analyses may be performed to explore the influence of study quality, sample characteristics, or intervention parameters on pooled effect sizes. The publication bias will be assessed using funnel plot visualization and statistically tested using Egger’s regression test, with adjustments considered, such as the trim-and-fill method, if asymmetry is detected. All meta- analysis will be performed in R-studio using *metaprop* function from the R-meta-package.

### Confidence in cumulative evidence

The certainty of evidence for each outcome will be evaluated using the GRADE framework [113]. This approach assesses the overall body of evidence across five key domains: risk of bias, inconsistency of results, indirectness of evidence, imprecision, and publication bias. Based on these criteria, evidence will be classified as high, moderate, low, or very low, reflecting the confidence in the results [113]. Employing the GRADE approach will ensure that the review not only synthesizes findings but also communicates the reliability of the evidence, which is essential for guiding culturally relevant interventions.

### Handling of missing data

For studies with missing, incomplete, or unclear information, attempts will be made to contact corresponding authors to obtain additional data or clarifications. When such data cannot be retrieved, the nature and extent of the missing information will be clearly documented. For quantitative outcomes, the potential impact of missing data on the results will be assessed, and sensitivity analyses will be performed where possible to evaluate how assumptions regarding missing data influence the findings. Studies with substantial or critical missing data that could undermine the validity of outcomes may be rated lower in quality or excluded from meta-analysis, though their results will still contribute to the narrative synthesis. The presence of missing data will also inform the risk of bias and quality assessments using the ROB2.0, ROBINS-I, and the STROBE checklist. By systematically addressing missing data, the review aims to reduce potential bias and ensure conclusions are based on the most complete and reliable evidence available.

### Patient and public involvement

Patients or the public were not involved in the design, conduct, reporting, or dissemination plans of our research.

### Ethical consideration

This study is a systematic review protocol that relies exclusively on previously published studies and secondary data sources. No new data will be collected from human participants or animals, and no interventions or experimental procedures will be conducted. As such, the review does not involve direct interaction with participants, nor does it include the collection of identifiable personal information. Consequently, ethical approval from an institutional review board or ethics committee was not required. The study will, however, adhere to established ethical standards for research conduct, including transparent reporting, accurate citation of original sources, and responsible synthesis of evidence. All included studies will be appropriately referenced to respect intellectual property and the integrity of the original research.

## Discussion

This review will synthesize current evidence on music-related cortical plasticity in adults. Music is a uniquely powerful stimulus, engaging auditory, visual, motor, cognitive, and emotional systems simultaneously. Converging research demonstrates that music can induce structural and functional brain changes [38,114], yet findings remain fragmented, with many studies addressing isolated outcomes such as neural activity [115,116], cerebral blood flow [44–46], or behavior [117,118]. What remains unclear is whether musical engagement induces coordinated structural, functional, and neurovascular brain plasticity in older adults, and whether these neural changes are associated with measurable improvements in cognitive and behavioral outcomes, including language, learning, and social functioning.

The primary aim of this review is to evaluate the evidence for music-induced neuroplasticity, encompassing structural adaptations (e.g., gray and white matter changes), functional dynamics (e.g., inter-regional connectivity and activity), and neurovascular responses (e.g., cerebral blood flow). A secondary aim is to link these neural changes to behavioral outcomes, including cognition, learning, and social functioning across adult populations (see **Fig 1**). This synthesis is clinically and socially relevant, as it may clarify whether and how music can serve as a tool to support brain health and well-being. Specifically, it may inform mental health interventions, optimize learning, and guide therapeutic approaches for aging populations at risk of cognitive decline.

Of note, the study has some strengths worth mentioning. The review will follow standard systematic review guidelines to ensure rigor and transparency. Multiple major scientific databases will be searched, supplemented by reference screening of prior reviews to enhance breath and coverage. Eligible studies will include randomized and non-randomized trials as well as observational designs. Study quality will be appraised using established tools. Anticipated challenges will include methodological heterogeneity, variable research quality, and the complexity of cultural, developmental, and music-type influences, which may constrain the ability to generalize findings or conduct meta-analysis.

In conclusion, upon successful completion, this review will advance understanding of how music influences neural and behavioral processes in adults by integrating evidence on its effects on brain structure, function, and cerebral hemodynamics. It will identify critical knowledge gaps and inform future research directions. The findings may support the development of evidence-based interventions to enhance cognition, strengthen emotional resilience, and mitigate age-related cognitive decline, thereby advancing the application of music as a therapeutic and preventive strategy for brain health.

## Supporting information

S1 File PRISMA-P (Preferred Reporting Items for Systematic Review and Meta-Analysis Protocols) 2015 checklist: Recommended items to address in a systematic review protocol. (DOCX)

## Abbreviation:

CBF: Cerebral Blood Flow
CENTRAL: Cochrane Central Register of Controlled Trials
DTI: Diffusion Tensor Imaging
EEG: Electroencephalography
EMBASE: Excerpta Medica Database
ERP: Event-Related Potential
GM: Gray Matter
MEG: Magnetoencephalography
MRI: Magnetic Resonance Imaging
NHLBI: National Heart, Lung, and Blood Institute
PRISMA: Preferred Reporting Items for Systematic Reviews and Meta-Analyses
PRISMA-P: Preferred Reporting Items for Systematic Review and Meta-Analysis Protocols
PROSPERO: International Prospective Register of Systematic Reviews
ROBINS-E: Risk of Bias in Non-randomized Studies – of Exposures
ROBINS-I: Risk of Bias in Non-randomized Studies – of Interventions
RoB 2.0: Risk of Bias 2.0
STROBE: Strengthening the Reporting of Observational Studies in Epidemiology
SWiM: Synthesis Without Meta-analysis
WM: White Matter

## References

[1] Gordon CL, Cobb PR, Balasubramaniam R. Recruitment of the motor system during music listening: an ALE meta-analysis of fMRI data. PLoS One. 2018;13(11):e0207213. doi: 10.1371/journal.pone.0207213 30452442 PMC6242316

[2] Lee H, Noppeney U. Long-term music training tunes how the brain temporally binds signals from multiple senses. Proc Natl Acad Sci U S A. 2011;108(51):E1441–50. doi: 10.1073/pnas.1115267108 22114191 PMC3251069

[3] Trost W, Trevor C, Fernandez N, Steiner F, Frühholz S. Live music stimulates the affective brain and emotionally entrains listeners in real time. Proc Natl Acad Sci U S A. 2024;121(10):e2316306121. doi: 10.1073/pnas.2316306121 38408255 PMC10927510

[4] Mongelli V, Dehaene S, Vinckier F, Peretz I, Bartolomeo P, Cohen L. Music and words in the visual cortex: the impact of musical expertise. Cortex. 2017;86:260–74. doi: 10.1016/j.cortex.2016.05.016 27317491

[5] Møller C, Garza-Villarreal EA, Hansen NC, Højlund A, Bærentsen KB, Chakravarty MM, et al. Audiovisual structural connectivity in musicians and non-musicians: a cortical thickness and diffusion tensor imaging study. Sci Rep. 2021;11(1):4324. doi: 10.1038/s41598-021-83135-x 33619288 PMC7900203

[6] Stegemöller EL. Exploring a neuroplasticity model of music therapy. J Music Ther. 2014;51(3):211–27. doi: 10.1093/jmt/thu023 25316915

[7] Schlaug G. Musicians and music making as a model for the study of brain plasticity. Prog Brain Res. 2015;217:37–55. doi: 10.1016/bs.pbr.2014.11.020 25725909 PMC4430083

[8] Chatterjee D, Hegde S, Thaut M. Neural plasticity: the substratum of music-based interventions in neurorehabilitation. NeuroRehabilitation. 2021;48(2):155–66. doi: 10.3233/NRE-208011 33579881 PMC7613141

[9] Münte TF, Altenmüller E, Jäncke L. The musician’s brain as a model of neuroplasticity. Nat Rev Neurosci. 2002;3(6):473–8. doi: 10.1038/nrn843 12042882

[10] Musliu A, Berisha B, Latifi D. The impact of music in memory. Eur J Soc Sci Educ Res. 2017;10(2):222. doi: 10.26417/ejser.v10i2.p222-227

[11] Snyder B. Music and memory: An introduction. MIT Press; 2000.

[12] Fernandez NB, Trost WJ, Vuilleumier P. Brain networks mediating the influence of background music on selective attention. Soc Cogn Affect Neurosci. 2019;14(12):1441–52. doi: 10.1093/scan/nsaa004 31993668 PMC7137722

[13] Jolij J, Meurs M. Music alters visual perception. PLoS One. 2011;6(4):e18861. doi: 10.1371/journal.pone.0018861 21533041 PMC3080883

[14] Wan CY, Schlaug G. Music making as a tool for promoting brain plasticity across the life span. Neuroscientist. 2010;16(5):566–77. doi: 10.1177/1073858410377805 20889966 PMC2996135

[15] Reybrouck M, Brattico E. Neuroplasticity beyond sounds: neural adaptations following long-term musical aesthetic experiences. Brain Sci. 2015;5(1):69–91. doi: 10.3390/brainsci5010069 25807006 PMC4390792

[16] Reybrouck M, Vuust P, Brattico E. Music and brain plasticity: how sounds trigger neurogenerative adaptations. Neuroplasticity - Insights of Neural Reorganization. InTech; 2018. pp. 85. doi: 10.5772/intechopen.74318

[17] Dalla Bella S. Music and brain plasticity. The Oxford handbook of music psychology. 2016. pp. 325–42.

[18] Olszewska AM, Gaca M, Herman AM, Jednoróg K, Marchewka A. How musical training shapes the adult brain: predispositions and neuroplasticity. Front Neurosci. 2021;15:630829. doi: 10.3389/fnins.2021.630829 33776638 PMC7987793

[19] Jaschke AC, Honing H, Scherder EJA. Exposure to a musically-enriched environment; Its relationship with executive functions, short-term memory and verbal IQ in primary school children. PLoS One. 2018;13(11):e0207265. doi: 10.1371/journal.pone.0207265 30419066 PMC6231655

[20] Bowmer A, Mason K, Knight J, Welch G. Investigating the impact of a musical intervention on preschool children’s executive function. Front Psychol. 2018;9:2389. doi: 10.3389/fpsyg.2018.02389 30618906 PMC6307457

[21] Mendes CG, de Paula JJ, Miranda DM. Effects of background music on attentional networks of children with and without attention deficit/hyperactivity disorder: case control experimental study. Interact J Med Res. 2024;13:e53869. doi: 10.2196/53869 39024557 PMC11294770

[22] Habibi A, Damasio A, Ilari B, Veiga R, Joshi AA, Leahy RM, et al. Childhood music training induces change in micro and macroscopic brain structure: results from a longitudinal study. Cereb Cortex. 2018;28(12):4336–47. doi: 10.1093/cercor/bhx286 29126181

[23] Särkämö T, Ripollés P, Vepsäläinen H, Autti T, Silvennoinen HM, Salli E, et al. Structural changes induced by daily music listening in the recovering brain after middle cerebral artery stroke: a voxel-based morphometry study. Front Hum Neurosci. 2014;8:245. doi: 10.3389/fnhum.2014.00245 24860466 PMC4029020

[24] Sa de Almeida J, Baud O, Fau S, Barcos-Munoz F, Courvoisier S, Lordier L, et al. Music impacts brain cortical microstructural maturation in very preterm infants: a longitudinal diffusion MR imaging study. Dev Cogn Neurosci. 2023;61:101254. doi: 10.1016/j.dcn.2023.101254 37182337 PMC10200857

[25] Haslbeck FB, Jakab A, Held U, Bassler D, Bucher H-U, Hagmann C. Creative music therapy to promote brain function and brain structure in preterm infants: a randomized controlled pilot study. Neuroimage Clin. 2020;25:102171. doi: 10.1016/j.nicl.2020.102171 31972397 PMC6974781

[26] Sihvonen AJ, Siponkoski S-T, Martínez-Molina N, Laitinen S, Holma M, Ahlfors M, et al. Neurological music therapy rebuilds structural connectome after traumatic brain injury: secondary analysis from a randomized controlled trial. J Clin Med. 2022;11(8):2184. doi: 10.3390/jcm11082184 35456277 PMC9032739

[27] Blood AJ, Zatorre RJ. Intensely pleasurable responses to music correlate with activity in brain regions implicated in reward and emotion. Proc Natl Acad Sci U S A. 2001;98(20):11818–23. doi: 10.1073/pnas.191355898 11573015 PMC58814

[28] Faßhauer C, Frese A, Evers S. Musical ability is associated with enhanced auditory and visual cognitive processing. BMC Neurosci. 2015;16:59. doi: 10.1186/s12868-015-0200-4 26377548 PMC4574220

[29] Meyer M, Elmer S, Ringli M, Oechslin MS, Baumann S, Jancke L. Long-term exposure to music enhances the sensitivity of the auditory system in children. Eur J Neurosci. 2011;34(5):755–65. doi: 10.1111/j.1460-9568.2011.07795.x 21848923

[30] Xu J, Yu L, Cai R, Zhang J, Sun X. Early auditory enrichment with music enhances auditory discrimination learning and alters NR2B protein expression in rat auditory cortex. Behav Brain Res. 2009;196(1):49–54. doi: 10.1016/j.bbr.2008.07.018 18706452

[31] Humphrey T. The effect of music ear training upon the auditory discrimination abilities of trainable mentally retarded adolescents. J Music Ther. 1980;17(2):70–4. doi: 10.1093/jmt/17.2.70 10247296

[32] Rochette F, Moussard A, Bigand E. Music lessons improve auditory perceptual and cognitive performance in deaf children. Front Hum Neurosci. 2014;8:488. doi: 10.3389/fnhum.2014.00488 25071518 PMC4076611

[33] Karpati FJ, Giacosa C, Foster NEV, Penhune VB, Hyde KL. Sensorimotor integration is enhanced in dancers and musicians. Exp Brain Res. 2016;234(3):893–903. doi: 10.1007/s00221-015-4524-1 26670906

[34] Hsu H-Y, Lin C-W, Lin Y-C, Wu P-T, Kato H, Su F-C, et al. Effects of vibrotactile-enhanced music-based intervention on sensorimotor control capacity in the hand of an aging brain: a pilot feasibility randomized crossover trial. BMC Geriatr. 2021;21(1):660. doi: 10.1186/s12877-021-02604-0 34814839 PMC8609800

[35] Carrer LRJ, Pompéia S, Miranda MC. Sensorimotor synchronization with music and metronome in school-aged children. Psychol Music. 2022;51(2):523–40. doi: 10.1177/03057356221100286

[36] Rodriguez-Gomez DA, Talero-Gutiérrez C. Effects of music training in executive function performance in children: a systematic review. Front Psychol. 2022;13:968144. doi: 10.3389/fpsyg.2022.968144 36003104 PMC9393548

[37] Luo C, Guo Z, Lai Y, Liao W, Liu Q, Kendrick KM, et al. Musical training induces functional plasticity in perceptual and motor networks: insights from resting-state FMRI. PLoS One. 2012;7(5):e36568. doi: 10.1371/journal.pone.0036568 22586478 PMC3346725

[38] Wu J, Zhang J, Ding X, Li R, Zhou C. The effects of music on brain functional networks: a network analysis. Neuroscience. 2013;250:49–59. doi: 10.1016/j.neuroscience.2013.06.021 23806719

[39] Alluri V, Toiviainen P, Burunat I, Kliuchko M, Vuust P, Brattico E. Connectivity patterns during music listening: evidence for action-based processing in musicians. Hum Brain Mapp. 2017;38(6):2955–70. doi: 10.1002/hbm.23565 28349620 PMC6866725

[40] Meyer M, Elmer S, Ringli M, Oechslin MS, Baumann S, Jancke L. Long-term exposure to music enhances the sensitivity of the auditory system in children. Eur J Neurosci. 2011;34(5):755–65. doi: 10.1111/j.1460-9568.2011.07795.x 21848923

[41] Nakamura S, Sadato N, Oohashi T, Nishina E, Fuwamoto Y, Yonekura Y. Analysis of music-brain interaction with simultaneous measurement of regional cerebral blood flow and electroencephalogram beta rhythm in human subjects. Neurosci Lett. 1999;275(3):222–6. doi: 10.1016/s0304-3940(99)00766-1 10580715

[42] Bigliassi M, Barreto-Silva V, Kanthack TFD, Altimari LR. Music and cortical blood flow: A functional near-infrared spectroscopy (fNIRS) study. Psychol Neurosci. 2014;7(4):545–50. doi: 10.3922/j.psns.2014.4.13

[43] Whiteford KL, Baltzell LS, Chiu M, Cooper JK, Faucher S, Goh PY, et al. Large-scale multi-site study shows no association between musical training and early auditory neural sound encoding. Nat Commun. 2025;16(1):7152. doi: 10.1038/s41467-025-62155-5 40781070 PMC12334757

[44] Kawasaki A, Hayashi N. Playing a musical instrument increases blood flow in the middle cerebral artery. PLoS One. 2022;17(6):e0269679. doi: 10.1371/journal.pone.0269679 35675278 PMC9176837

[45] Cavieres R, Landerretche J, Jara JL, Chacón M. Analysis of cerebral blood flow entropy while listening to music with emotional content. Physiol Meas. 2021;42(5). doi: 10.1088/1361-6579/abf885 33857935

[46] Ayaz A, Rahimi A, Buwadi L, Wang Y-B, Zou L, Heath M. Rocking the cerebral blood flow: the influence of music listening and aerobic exercise on cortical hemodynamics and post-intervention executive function. Exp Brain Res. 2025;243(4):102. doi: 10.1007/s00221-025-07054-3 40131455

[47] Paulson OB, Hasselbalch SG, Rostrup E, Knudsen GM, Pelligrino D. Cerebral blood flow response to functional activation. J Cereb Blood Flow Metab. 2010;30(1):2–14. doi: 10.1038/jcbfm.2009.188 19738630 PMC2872188

[48] Lauritzen M. Relationship of spikes, synaptic activity, and local changes of cerebral blood flow. J Cereb Blood Flow Metab. 2001;21(12):1367–83. doi: 10.1097/00004647-200112000-00001 11740198

[49] Blood AJ, Zatorre RJ, Bermudez P, Evans AC. Emotional responses to pleasant and unpleasant music correlate with activity in paralimbic brain regions. Nat Neurosci. 1999;2(4):382–7. doi: 10.1038/7299 10204547

[50] Altenmüller E, Marco-Pallares J, Münte TF, Schneider S. Neural reorganization underlies improvement in stroke-induced motor dysfunction by music-supported therapy. Ann N Y Acad Sci. 2009;1169:395–405. doi: 10.1111/j.1749-6632.2009.04580.x 19673814

[51] Chan AS, Ho YC, Cheung MC. Music training improves verbal memory. Nature. 1998;396(6707):128. doi: 10.1038/24075 9823892

[52] Franklin MS, Sledge Moore K, Yip C-Y, Jonides J, Rattray K, Moher J. The effects of musical training on verbal memory. Psychol Music. 2008;36(3):353–65. doi: 10.1177/0305735607086044

[53] Taylor AC, Dewhurst SA. Investigating the influence of music training on verbal memory. Psychol Music. 2017;45(6):814–20. doi: 10.1177/0305735617690246

[54] Kasuya-Ueba Y, Zhao S, Toichi M. The effect of music intervention on attention in children: experimental evidence. Front Neurosci. 2020;14:757. doi: 10.3389/fnins.2020.00757 32792900 PMC7393235

[55] Pasiali V, LaGasse AB, Penn SL. The effect of musical attention control training (MACT) on attention skills of adolescents with neurodevelopmental delays: a pilot study. J Music Ther. 2014;51(4):333–54. doi: 10.1093/jmt/thu030 25504177

[56] Grassi M, Talamini F, Altoè G, Brattico E, Caclin A, Carretti B, et al. Do musicians have better short-term memory than nonmusicians? A multilab study. Adv Methods Pract Psychol Sci. 2025;8(4). doi: 10.1177/25152459251379432

[57] Moore KS. A systematic review on the neural effects of music on emotion regulation: implications for music therapy practice. J Music Ther. 2013;50(3):198–242. doi: 10.1093/jmt/50.3.198 24568004

[58] Cook T, Roy ARK, Welker KM. Music as an emotion regulation strategy: an examination of genres of music and their roles in emotion regulation. Psychol Music. 2017;47(1):144–54. doi: 10.1177/0305735617734627

[59] Sharda M, Tuerk C, Chowdhury R, Jamey K, Foster N, Custo-Blanch M, et al. Music improves social communication and auditory-motor connectivity in children with autism. Transl Psychiatry. 2018;8(1):231. doi: 10.1038/s41398-018-0287-3 30352997 PMC6199253

[60] Boster JB, Spitzley AM, Castle TW, Jewell AR, Corso CL, McCarthy JW. Music improves social and participation outcomes for individuals with communication disorders: a systematic review. J Music Ther. 2021;58(1):12–42. doi: 10.1093/jmt/thaa015 32978945

[61] Bernatzky G, Bernatzky P, Hesse H-P, Staffen W, Ladurner G. Stimulating music increases motor coordination in patients afflicted with Morbus Parkinson. Neurosci Lett. 2004;361(1–3):4–8. doi: 10.1016/j.neulet.2003.12.022 15135879

[62] Arnaud C, Perlovsky L, Bonniot-Cabanac M-C, Cabanac M. Music and academic performance. Behav Brain Res. 2013;256:257–60. doi: 10.1016/j.bbr.2013.08.023 23973386

[63] Román-Caballero R, Arnedo M, Triviño M, Lupiáñez J. Musical practice as an enhancer of cognitive function in healthy aging - a systematic review and meta-analysis. PLoS One. 2018;13(11):e0207957. doi: 10.1371/journal.pone.0207957 30481227 PMC6258526

[64] Innes KE, Selfe TK, Khalsa DS, Kandati S. Meditation and music improve memory and cognitive function in adults with subjective cognitive decline: a pilot randomized controlled trial. J Alzheimers Dis. 2017;56(3):899–916. doi: 10.3233/JAD-160867 28106552 PMC7967907

[65] Hyde KL, Lerch J, Norton A, Forgeard M, Winner E, Evans AC, et al. The effects of musical training on structural brain development. Ann N Y Acad Sci. 2009;1169(1):182–6. doi: 10.1111/j.1749-6632.2009.04852.x 19673777

[66] Fischer CE, Churchill N, Leggieri M, Vuong V, Tau M, Fornazzari LR, et al. Long-known music exposure effects on brain imaging and cognition in early-stage cognitive decline: a pilot study. J Alzheimers Dis. 2021;84(2):819–33. doi: 10.3233/JAD-210610 34602475

[67] Gaser C, Schlaug G. Brain structures differ between musicians and non-musicians. J Neurosci. 2003;23(27):9240–5. doi: 10.1523/JNEUROSCI.23-27-09240.2003 14534258 PMC6740845

[68] Zhang L, Peng W, Chen J, Hu L. Electrophysiological evidences demonstrating differences in brain functions between nonmusicians and musicians. Sci Rep. 2015;5:13796. doi: 10.1038/srep13796 26338509 PMC4559803

[69] Schmithorst VJ, Wilke M. Differences in white matter architecture between musicians and non-musicians: a diffusion tensor imaging study. Neurosci Lett. 2002;321(1–2):57–60. doi: 10.1016/s0304-3940(02)00054-x 11872256

[70] Zuk J, Benjamin C, Kenyon A, Gaab N. Behavioral and neural correlates of executive functioning in musicians and non-musicians. PLoS One. 2014;9(6):e99868. doi: 10.1371/journal.pone.0099868 24937544 PMC4061064

[71] Schulze K, Mueller K, Koelsch S. Neural correlates of strategy use during auditory working memory in musicians and non-musicians. Eur J Neurosci. 2011;33(1):189–96. doi: 10.1111/j.1460-9568.2010.07470.x 21073548

[72] Partanen E, Kujala T, Tervaniemi M, Huotilainen M. Prenatal music exposure induces long-term neural effects. PLoS One. 2013;8(10):e78946. doi: 10.1371/journal.pone.0078946 24205353 PMC3813619

[73] Mahmood D, Nisar H, Yap VV, Tsai C-Y. The effect of music listening on EEG functional connectivity of brain: a short-duration and long-duration study. Mathematics. 2022;10(3):349. doi: 10.3390/math10030349

[74] Raglio A, Galandra C, Sibilla L, Esposito F, Gaeta F, Di Salle F, et al. Effects of active music therapy on the normal brain: fMRI based evidence. Brain Imaging Behav. 2016;10(1):182–6. doi: 10.1007/s11682-015-9380-x 25847861

[75] Steinhoff N, Heine AM, Vogl J, Weiss K, Aschraf A, Hajek P, et al. A pilot study into the effects of music therapy on different areas of the brain of individuals with unresponsive wakefulness syndrome. Front Neurosci. 2015;9:291. doi: 10.3389/fnins.2015.00291 26347603 PMC4543917

[76] Lanzilotti C, Dumas R, Grassi M, Schön D. Prolonged exposure to highly rhythmic music affects brain dynamics and perception. Neuropsychologia. 2019;129:191–9. doi: 10.1016/j.neuropsychologia.2019.04.011 31015025

[77] Xiao X, Chen W, Zhang X. The effect and mechanisms of music therapy on the autonomic nervous system and brain networks of patients of minimal conscious states: a randomized controlled trial. Front Neurosci. 2023;17:1182181. doi: 10.3389/fnins.2023.1182181 37250411 PMC10213399

[78] Moore E, Schaefer RS, Bastin ME, Roberts N, Overy K. Diffusion tensor MRI tractography reveals increased fractional anisotropy (FA) in arcuate fasciculus following music-cued motor training. Brain Cogn. 2017;116:40–6. doi: 10.1016/j.bandc.2017.05.001 28618361 PMC5479403

[79] Fachner J, Gold C, Erkkilä J. Music therapy modulates fronto-temporal activity in rest-EEG in depressed clients. Brain Topogr. 2013;26(2):338–54. doi: 10.1007/s10548-012-0254-x 22983820

[80] Bhattacharya J, Lee E-J. Modulation of EEG theta band signal complexity by music therapy. Int J Bifurcation Chaos. 2016;26(01):1650001. doi: 10.1142/s0218127416500012

[81] Nawaz R, Nisar H, Voon YV. The effect of music on human brain; frequency domain and time series analysis using electroencephalogram. IEEE Access. 2018;6:45191–205. doi: 10.1109/access.2018.2855194

[82] Hauck M, Metzner S, Rohlffs F, Lorenz J, Engel AK. The influence of music and music therapy on pain-induced neuronal oscillations measured by magnetencephalography. Pain. 2013;154(4):539–47. doi: 10.1016/j.pain.2012.12.016 23414577

[83] Buard I, Dewispelaere WB, Thaut M, Kluger BM. Preliminary neurophysiological evidence of altered cortical activity and connectivity with neurologic music therapy in Parkinson’s disease. Front Neurosci. 2019;13:105. doi: 10.3389/fnins.2019.00105 30837830 PMC6390231

[84] Fujioka T, Chen JL, Black SE, Chen JJ, Honjo K, Dawson DR, et al. Beta- and gamma-band neuromagnetic oscillations in chronic stroke rehabilitation using music-supported therapy and manual training. Ann N Y Acad Sci. 2025;1552(1):362–72. doi: 10.1111/nyas.70041 40930509

[85] Rajakumar KD, Mohan J. A systematic review on effect of music intervention on cognitive impairment using EEG, fMRI, and cognitive assessment modalities. Result Eng. 2024;22:102224. doi: 10.1016/j.rineng.2024.102224

[86] Feng K, Shen C-Y, Ma X-Y, Chen G-F, Zhang M-L, Xu B, et al. Effects of music therapy on major depressive disorder: A study of prefrontal hemodynamic functions using fNIRS. Psychiatry Res. 2019;275:86–93. doi: 10.1016/j.psychres.2019.03.015 30884335

[87] Gutiérrez Santamaría S. Microstructural changes related to dementia and music therapy in memory impaired patients measured with diffusion MRI: first insights of the ALMUTH trial. 2022.

[88] Siponkoski S-T, Martínez-Molina N, Kuusela L, Laitinen S, Holma M, Ahlfors M, et al. Music therapy enhances executive functions and prefrontal structural neuroplasticity after traumatic brain injury: evidence from a randomized controlled trial. J Neurotrauma. 2020;37(4):618–34. doi: 10.1089/neu.2019.6413 31642408

[89] Braz CH, Gonçalves LF, Paiva KM, Haas P, Patatt FSA. Implications of musical practice in central auditory processing: a systematic review. Braz J Otorhinolaryngol. 2021;87(2):217–26. doi: 10.1016/j.bjorl.2020.10.007 33309194 PMC9422430

[90] Rauschecker JP. Cortical plasticity and music. Ann N Y Acad Sci. 2001;930:330–6. doi: 10.1111/j.1749-6632.2001.tb05742.x 11458839

[91] Abdul-Kareem IA, Stancak A, Parkes LM, Sluming V. Increased gray matter volume of left pars opercularis in male orchestral musicians correlate positively with years of musical performance. J Magn Reson Imaging. 2011;33(1):24–32. doi: 10.1002/jmri.22391 21182117

[92] Acer N, Bastepe-Gray S, Sagiroglu A, Gumus KZ, Degirmencioglu L, Zararsiz G, et al. Diffusion tensor and volumetric magnetic resonance imaging findings in the brains of professional musicians. J Chem Neuroanat. 2018;88:33–40. doi: 10.1016/j.jchemneu.2017.11.003 29113947

[93] Alfredson BB, Risberg J, Hagberg B, Gustafson L. Right temporal lobe activation when listening to emotionally significant music. Appl Neuropsychol. 2004;11(3):161–6. doi: 10.1207/s15324826an1103_4 15590350

[94] Acer N, Kamaşak Arpaçay B, Bastepe Gray S, Oğuzhan Karapınar B, İpekten F, Değirmencioğlu L, et al. Structural and functional changes in the brains of guitarist musicians: volumetric, VBM, and resting state fMRI study. J Clin Pract Res. 2024;46(1):47–57. doi: 10.14744/cpr.2024.98608 41255904 PMC12478835

[95] Heller W. Neuropsychological mechanisms of individual differences in emotion, personality, and arousal. Neuropsychology. 1993;7(4):476–89. doi: 10.1037/0894-4105.7.4.476

[96] Rossi AF, Pessoa L, Desimone R, Ungerleider LG. The prefrontal cortex and the executive control of attention. Exp Brain Res. 2009;192(3):489–97. doi: 10.1007/s00221-008-1642-z 19030851 PMC2752881

[97] Kaiser AP, Berntsen D. The cognitive characteristics of music-evoked autobiographical memories: Evidence from a systematic review of clinical investigations. Wiley Interdiscip Rev Cogn Sci. 2023;14(3):e1627. doi: 10.1002/wcs.1627 36223919

[98] Talamini F, Altoè G, Carretti B, Grassi M. Musicians have better memory than nonmusicians: a meta-analysis. PLoS One. 2017;12(10):e0186773. doi: 10.1371/journal.pone.0186773 29049416 PMC5648224

[99] Martin-Moratinos M, Bella-Fernández M, Blasco-Fontecilla H. Effects of music on attention-deficit/hyperactivity disorder (ADHD) and potential application in serious video games: systematic review. J Med Internet Res. 2023;25:e37742. doi: 10.2196/37742 37171837 PMC10221503

[100] Chee ZJ, Chang CYM, Cheong JY, Malek FHBA, Hussain S, de Vries M, et al. The effects of music and auditory stimulation on autonomic arousal, cognition and attention: a systematic review. Int J Psychophysiol. 2024;199:112328. doi: 10.1016/j.ijpsycho.2024.112328 38458383

[101] Liu Q, Li W, Yin Y, Zhao Z, Yang Y, Zhao Y, et al. The effect of music therapy on language recovery in patients with aphasia after stroke: a systematic review and meta-analysis. Neurol Sci. 2022;43(2):863–72. doi: 10.1007/s10072-021-05743-9 34816318

[102] Yang Y, Fang Y-Y, Gao J, Geng G-L. Effects of five-element music on language recovery in patients with poststroke aphasia: a systematic review and meta-analysis. J Altern Complement Med. 2019;25(10):993–1004. doi: 10.1089/acm.2018.0479 31298550

[103] Uhlig S, Jaschke A, Scherder E. Effects of music on emotion regulation: A systematic literature review. 2013.

[104] Moher D, Shamseer L, Clarke M, Ghersi D, Liberati A, Petticrew M, et al. Preferred reporting items for systematic review and meta-analysis protocols (PRISMA-P) 2015 statement. Syst Rev. 2015;4(1):1. doi: 10.1186/2046-4053-4-1 25554246 PMC4320440

[105] Shamseer L, Moher D, Clarke M, Ghersi D, Liberati A, Petticrew M, et al. Preferred reporting items for systematic review and meta-analysis protocols (PRISMA-P) 2015: elaboration and explanation. BMJ. 2015;349(jan02 1):g7647. doi: 10.1136/bmj.g7647 25555855

[106] Page MJ, McKenzie JE, Bossuyt PM, Boutron I, Hoffmann TC, Mulrow CD, et al. The PRISMA 2020 statement: an updated guideline for reporting systematic reviews. BMJ. 2021;372:n71. doi: 10.1136/bmj.n71 33782057 PMC8005924

[107] Campbell M, McKenzie JE, Sowden A, Katikireddi SV, Brennan SE, Ellis S, et al. Synthesis without meta-analysis (SWiM) in systematic reviews: reporting guideline. BMJ. 2020;368:l6890. doi: 10.1136/bmj.l6890 31948937 PMC7190266

[108] Minozzi S, Cinquini M, Gianola S, Gonzalez-Lorenzo M, Banzi R. The revised Cochrane risk of bias tool for randomized trials (RoB 2) showed low interrater reliability and challenges in its application. J Clin Epidemiol. 2020;126:37–44. doi: 10.1016/j.jclinepi.2020.06.015 32562833

[109] Jüni P, Loke Y, Pigott T, Ramsay C, Regidor D, Rothstein H, et al. Risk of bias in non-randomized studies of interventions (ROBINS-I): detailed guidance. Br Med J. 2016;355:i4919.

[110] Higgins JPT, Morgan RL, Rooney AA, Taylor KW, Thayer KA, Silva RA, et al. A tool to assess risk of bias in non-randomized follow-up studies of exposure effects (ROBINS-E). Environ Int. 2024;186:108602. doi: 10.1016/j.envint.2024.108602 38555664 PMC11098530

[111] Cevallos M, Egger M. STROBE (STrengthening the Reporting of OBservational studies in Epidemiology). Guidelines for reporting health research: a user’s manual. 2014. pp. 169–79.

[112] Page MJ, McKenzie JE, Bossuyt PM, Boutron I, Hoffmann TC, Mulrow CD, et al. The PRISMA 2020 statement: an updated guideline for reporting systematic reviews. BMJ. 2021;372:n71. doi: 10.1136/bmj.n71 33782057 PMC8005924

[113] Chi C-C, Shao S-C, Kuo L-T, Huang Y-T, Lai P-C. Using Grading of Recommendations Assessment, Development, and Evaluation (GRADE) to rate the certainty of evidence of study outcomes from systematic reviews: A quick tutorial. Dermatol Sin. 2023;41(1):3. doi: 10.4103/ds.ds-d-22-00154

[114] Toader C, Tataru CP, Florian I-A, Covache-Busuioc R-A, Bratu B-G, Glavan LA, et al. Cognitive crescendo: how music shapes the brain’s structure and function. Brain Sci. 2023;13(10):1390. doi: 10.3390/brainsci13101390 37891759 PMC10605363

[115] Creutzfeldt O, Ojemann G. Neuronal activity in the human lateral temporal lobe. III. Activity changes during music. Exp Brain Res. 1989;77(3):490–8. doi: 10.1007/BF00249602 2806443

[116] Freitas C, Manzato E, Burini A, Taylor MJ, Lerch JP, Anagnostou E. Neural correlates of familiarity in music listening: a systematic review and a neuroimaging meta-analysis. Front Neurosci. 2018;12:686. doi: 10.3389/fnins.2018.00686 30344470 PMC6183416

[117] Zhang Y, Cai J, An L, Hui F, Ren T, Ma H, et al. Does music therapy enhance behavioral and cognitive function in elderly dementia patients? A systematic review and meta-analysis. Ageing Res Rev. 2017;35:1–11. doi: 10.1016/j.arr.2016.12.003 28025173

[118] Ueda T, Suzukamo Y, Sato M, Izumi S-I. Effects of music therapy on behavioral and psychological symptoms of dementia: a systematic review and meta-analysis. Ageing Res Rev. 2013;12(2):628–41. doi: 10.1016/j.arr.2013.02.003 23511664

